# Nuclear RNA Exosome and Pervasive Transcription: Dual Sculptors of Genome Function

**DOI:** 10.3390/ijms222413401

**Published:** 2021-12-13

**Authors:** Koichi Ogami, Hiroshi I. Suzuki

**Affiliations:** 1 Division of Molecular Oncology, Center for Neurological Diseases and Cancer, Nagoya University Graduate School of Medicine, 65 Tsurumai-cho, Showa-ku, Nagoya 466-8550, Japan; koichi.ogami@med.nagoya-u.ac.jp; 2 Institute for Glyco-core Research (iGCORE), Nagoya University, Furo-cho, Chikusa-ku, Nagoya 464-8601, Japan

**Keywords:** RNA exosome, pervasive transcription, cofactor, genome, evolution, disease

## Abstract

The genome is pervasively transcribed across various species, yielding numerous non-coding RNAs. As a counterbalance for pervasive transcription, various organisms have a nuclear RNA exosome complex, whose structure is well conserved between yeast and mammalian cells. The RNA exosome not only regulates the processing of stable RNA species, such as rRNAs, tRNAs, small nucleolar RNAs, and small nuclear RNAs, but also plays a central role in RNA surveillance by degrading many unstable RNAs and misprocessed pre-mRNAs. In addition, associated cofactors of RNA exosome direct the exosome to distinct classes of RNA substrates, suggesting divergent and/or multi-layer control of RNA quality in the cell. While the RNA exosome is essential for cell viability and influences various cellular processes, mutations and alterations in the RNA exosome components are linked to the collection of rare diseases and various diseases including cancer, respectively. The present review summarizes the relationships between pervasive transcription and RNA exosome, including evolutionary crosstalk, mechanisms of RNA exosome-mediated RNA surveillance, and physiopathological effects of perturbation of RNA exosome.

## 1. Introduction

Precise spatio-temporal control of gene expression is critical for converting genomic information into biological functions of life systems. Transcription forms an initial foundation of the gene expression programs; eukaryotic genomes, including those of yeasts, are pervasively transcribed beyond the canonical protein-coding genes and representative non-coding RNA genes, such as rRNAs, yielding numerous RNA species such as long non-coding RNAs (lncRNAs), upstream antisense RNAs (uaRNAs, also known as promoter upstream transcripts (PROMPTs) in mammals or cryptic unstable transcripts (CUTs) in yeasts), transcription start site (TSS)-associated RNAs (tssRNAs), and enhancer RNAs (eRNAs) [1,2]. Typically, these RNA species are unstable and subject to rapid degradation by the RNA exosome complex, a counterbalance system for pervasive transcription. The RNA exosome not only regulates the processing of stable RNA species such as rRNAs, tRNAs, small nucleolar RNAs (snoRNAs), and small nuclear RNAs (snRNAs), but also plays a central role in RNA surveillance by degrading many unstable RNAs and misprocessed pre-mRNAs [3,4]. Besides these “classical” exosome substrates, the spectrum of exosome target transcripts is still expanding. In addition, associated cofactors of RNA exosome direct the exosome to distinct classes of RNA substrates, suggesting divergent and/or multi-layer control of RNA quality in the cell [3,4,5]. The present review summarizes the relationships between pervasive transcription and RNA exosome, including evolutionary crosstalk, mechanisms of RNA exosome-mediated RNA surveillance, and physiopathological effects of perturbation of RNA exosome. While the RNA exosome has been well studied in various species including yeasts and flies, this review mainly focuses on the findings in mammalian cells.

## 2. Molecular Mechanisms of RNA Surveillance by RNA Exosome

### 2.1. Structure of RNA Exosome

**Figure 1:**
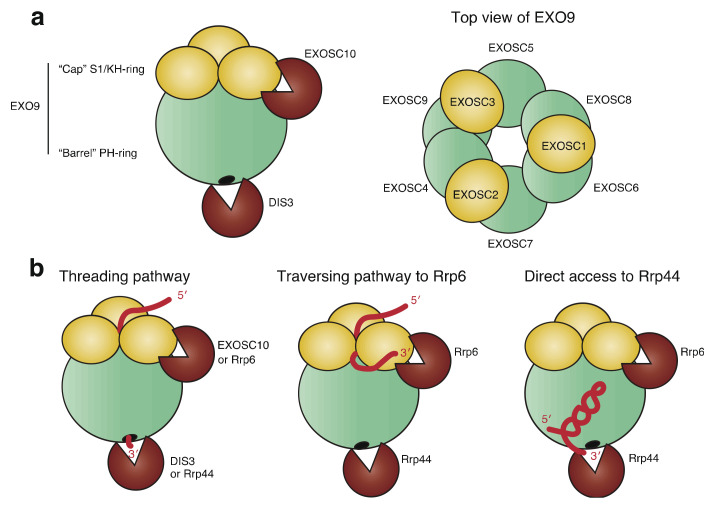
Structure of RNA exosome and pathways leading RNA substrates to the catalytic subunits. (**a**) The core RNA exosome (EXO9) is composed of nine subunits and is further divided into two structures, “cap” S1/KH ring and “barrel” PH-ring. Two catalytic subunits EXOSC10 and DIS3 are placed near the cap and at the bottom of barrel, respectively. (**b**) Three distinct pathways identified so far, which lead RNA 3′ end to either of the two catalytic subunits. Threading pathway: RNA is threaded into the central channel of the core exosome and brought to the active site of DIS3; Traversing pathway to Rrp6: RNA traverses the cap structure, reaching the active site of Rrp6; and Direct access to Rrp44: RNA directly accesses Rrp44 without entering the central channel.

The structure and composition of RNA exosome are well conserved among eukaryotes and archaea, and a similar complex has been identified in bacteria [6]. The human nuclear RNA exosome consists of a catalytically inactive core complex (EXO9) with nine proteins and two catalytic subunits, EXOSC10 (also known as rRNA-processing protein 6 (Rrp6) or PM/Scl-100) and DIS3 (also known as Rrp44 or EXOSC11) (Figure 1) [7]. The barrel-shaped core complex can further be divided into two characteristic complexes: a cap structure comprising three S1/K homology (KH) proteins (EXOSC1-3, also known as Rrp4, Rrp40, and Csl4) (“S1/KH cap”) and a ring structure comprising six RNase pleckstrin homology (PH)-like proteins (EXOSC4-9, also known as Rrp41, Rrp42, Rrp43, Rrp45, Rrp46, and Mtr3) (“PH ring or barrel”) (Figure 1a) [7]. The top of the S1/KH cap is an RNA entry pore, and the bottom of the PH barrel is an RNA exit pore. EXOSC10 and DIS3 are placed near the RNA entry and at the exit pore, respectively (Figure 1a). RNA substrates can either be degraded or 3′ trimmed by EXOSC10 or DIS3. Three distinct pathways leading an RNA to either of these proteins were discovered in budding yeast (Figure 1b) [8,9,10,11,12,13,14]. The threading pathway, in which an RNA is threaded into the central channel of the core complex in a 3′-5′ orientation, depends on Rrp44 (the yeast homolog of DIS3) at the exit pore to degrade the RNA. In the traversing pathway, RNA enters the S1/KH cap ring, but, instead of threading into the barrel, the 3′ end of RNA traverses the cap. Rrp6 (the yeast homolog of EXOSC10) at the top of the cap is responsible for the degradation or trimming of the traversed RNA. The third pathway is the direct access of RNA to Rrp44 without entering the core structure. Recent studies using cryogenic electron microscopy (cryo-EM) have also successfully captured the structure of the threading human nuclear exosome [15,16]. It remains unclear whether the traversing and direct pathways exist in the human exosome system.

A recent proteomic analysis of cell cycle-dependent protein interactions suggests that the dynamics of EXOSC components can be divided into three groups: (1) PH ring components (EXOSC4–9) and EXOSC2, (2) EXOSC1 and EXOSC3, and (3) EXOSC10 [17]. EXOSC1, EXOSC3, and EXOSC10 are less stably associated with the core exosome complex than the other EXO9 subunits, possibly suggesting that these proteins have independent functions outside of the exosome complex [18,19]. Indeed, the exosome-independent functions of EXOSC1 have been suggested [19].

### 2.2. Sorting of Target RNAs by Adaptors and Cofactors of RNA Exosome

**Figure 2:**
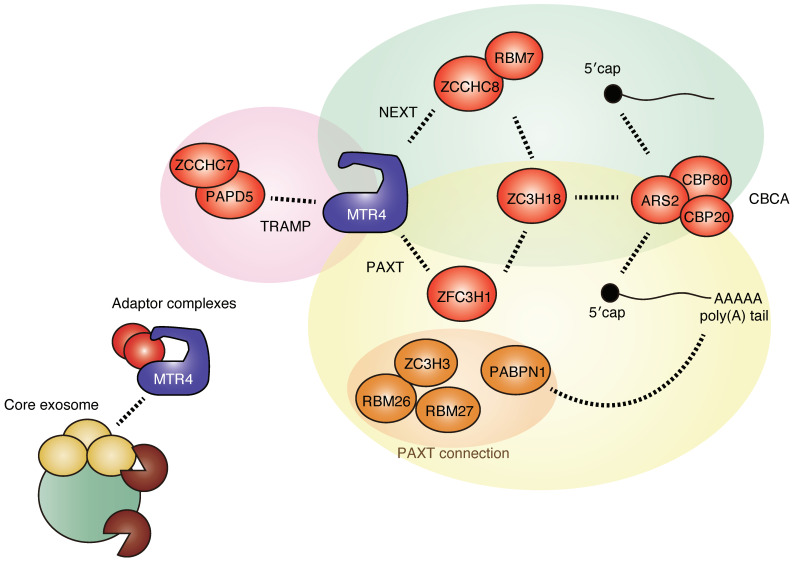
The human adaptor complexes guiding specific RNA substrates for exosome-mediated RNA degradation or processing. Note that the RNA helicase MTR4 is commonly present in all the adaptor complexes.

While the RNA exosome can degrade most RNA per se, multiple distinct adaptor complexes have been shown to direct the RNA exosome to distinct classes of RNA substrates (Figure 2). In most organisms, the RNA exosome has distinct partners in the nucleolus and nucleoplasm, although the Trf4/5-Air1/2-Mtr4 polyadenylation (TRAMP) complex plays a predominant role in both the nucleolus and nucleoplasm in *Saccharomyces cerevisiae*. In humans, the TRAMP complex consists of MTR4 (also known as SKIV2L2 or MTREX) and human homologs of the yeast Trf4/5 and Air1/2, non-canonical poly(A) polymerase PAPD5 (also known as hTrf4-2 or TENT4B) and ZCCHC7, respectively [20]. In contrast to budding yeast, the human TRAMP is restricted to the nucleolus and is involved in rRNA processing [20].

In addition to the TRAMP complex, additional nucleoplasmic adaptor complexes have been identified in mammals. The nuclear exosome targeting complex (NEXT) and the poly(A) tail exosome targeting complex (PAXT) remove various types of nucleoplasmic lncRNAs, and many components of these complexes are conserved in *Schizosaccharomyces pombe*, especially PAXT. The NEXT complex consists of MTR4, the RNA-binding protein RBM7, the zinc-finger protein ZCCHC8, and degrades snRNA, snoRNA, replication-dependent histone mRNAs, uaRNAs, eRNAs, and LINE1 RNAs [20,21,22]. The PAXT complex consists of MTR4 and the zing-finger protein ZFC3H1 and functions in cooperation with the nuclear poly(A)-binding protein PABPN1 [23]. Three additional RNA-binding proteins, ZC3H3, RBM26, and RBM27, are required for the full activity of PAXT [24]. The PAXT complex targets snoRNA host gene (SNHG) transcripts, pre-mRNAs cleaved and polyadenylated in an intron (prematurely terminated RNAs; ptRNAs), uaRNAs, and eRNAs [23,25]. While the targets of NEXT and PAXT overlap, PAXT targets tend to be longer and more extensively polyadenylated than NEXT targets [23]. Promiscuous RNA binding by RBM7 with some preferences for U-rich pyrimidine stretches and recognition of poly(A) tails by PABPN1 appear to have important roles in target selection by NEXT and PAXT, respectively [22,23]. In addition, the connection between NEXT/PAXT and the cap-binding complex CBCA, which consists of cap-binding proteins CBP20 (also known as NCBP2), CBP80 (also known as NCBP1), and ARS2 (also known as SRRT) with ZC3H18, supports the physical interaction between NEXT/PAXT and capped RNAs [20,23,26,27]. Notably, MTR4 is present in all other adaptor complexes and thus plays a central role in complex assembly and RNA sorting.

A recent report has shown that NEXT targets become polyadenylated upon NEXT inhibition and are handed over to PAXT-mediated decay, indicating fail-safe RNA decay via PAXT [28]. This may be consistent with the deep conservation of PAXT components from fission yeast to humans, relative to NEXT components. Given that NEXT targets non-polyadenylated RNA 3′ ends distributed over kilobase-wide regions [28], NEXT may be adapted for the quality control of transcription of longer genes in higher organisms, as gene length in mammals is longer than that in yeast.

### 2.3. Balance between RNA Export and Degradation

CBCA (especially ARS2) plays an important role in determining whether target RNAs are subjected to productive processing and export or to nuclear retention and decay [29]. CBCA-bound mRNAs and snRNA/snoRNAs are directed toward productive processing by CBCA-associated nuclear export factor ALYREF and phosphorylated adaptor for RNA export (PHAX), respectively. ZC3H18, a CBCA-NEXT and CBCA-PAXT partner, physically competes with PHAX for CBCA binding, and these proteins oppositely define the fate of RNAs: degradation or export [23,30]. PHAX and ZC3H18 only transiently bind to CBC (CBP20/CBP80) and exchange rapidly and continuously on the CBC during transcription, allowing determination of the fate of RNAs at particular checkpoints [30]. As for replication-dependent histone mRNA, CBCA-associated FLASH commits these transcripts for maturation [31]. Similar to other exosome targets, replication-dependent histone mRNAs accumulate upon ARS2 depletion [26,32]; however, the competition between FLASH and the exosome adaptors for CBCA binding has not been experimentally examined.

Recent studies have revealed that PAXT plays an important role in balancing RNA degradation and export. Depletion of MTR4 and ZFC3H1 has been shown to cause accumulation of uaRNAs and ptRNAs not only in the nucleoplasm but also in the cytoplasm, resulting in cytoplasmic accumulation of abnormally ribosome-bound RNAs [25,33]. One explanation for the cytoplasmic leakage of PAXT targets observed under PAXT depletion is that MTR4 physically competes with ALYREF for association with ARS2 [34]. Importantly, compartmentalized distribution of RNAs seems to be a key to understanding RNA degradation-export balancing. When the nuclear exosome is depleted, the excessive formation of polyA+ RNA foci in the nucleus is reproducibly observed [34,35]. Although the full composition of the polyA+ RNA foci is yet to be determined, it at least includes all the PAXT components as well as SNHG RNAs and exosome target mRNAs [34,35]. The formation of the foci depends on the condensation activity of ZFC3H1 through its N-terminal intrinsically disordered region [36], and the depletion of ZFC3H1 results in disruption of the nuclear polyA+ foci formed upon exosome inactivation [35,36]. Therefore, ZFC3H1 functions not only as a degradation factor but also as a nuclear RNA retention factor.

An additional important factor is the MTR4-binding partner NRDE2. NRDE2 interacts with MTR4 in a 1:1 stoichiometry, but the complex is devoid of the core exosome complex and is independent of the other adaptor complexes [37,38]. NRDE2 is enriched in nuclear speckles co-localized with polyA RNAs and represses exosome-mediated RNA degradation [38]. NRDE2 knockdown also affects RNA export, which is not due to the suggested intron retention, indicating that NRDE2-containing speckles are additional layers that control the balance between RNA degradation and export [38].

## 3. Evolutionary Interplays between RNA Exosome and Pervasive Transcription

How many of the unstable transcripts targeted by RNA exosome have biological functions? An ostensibly wasteful process of pervasive transcription and subsequent degradation by the RNA exosome may be reconciled with their evolutionary interplay. A recent review of lncRNAs provides insights into this issue [39]. In contrast to coding sequences, less than 10% of human genomic DNA is estimated to evolve under purifying selection [39]. Thus, the majority of genomic targets of pervasive transcription are considered to have evolved neutrally. In fact, overall, lncRNAs are considerably less conserved than untranslated regions (UTRs) of mRNAs, suggesting an inherent plastic nature [40]. In the evolutionary scenario, where eukaryotes evolve under a weak selection regime with genetic drift, it is unlikely that lncRNAs are maintained by positive selection in general. In the weak selection regime, promiscuity of the eukaryotic transcription machinery, including divergent transcription by RNA polymerase II and recognition of short and degenerative motifs by transcription factors, yields numerous RNA species without conserved functions, as even most of the heterochromatin is also transcribed [1,41,42]. The population genetic standpoint raises the hypothesis that organisms with small effective population size evolve the RNA exosome system as global solutions to deal with local problems, i.e., mutations that create a cryptic transcript; otherwise, the deleterious effects of transcription-inducing mutations cannot be efficiently eliminated from the population in the weak selection regime [39]. Global repression of unstable transcripts but allowance for low expression is thought to leave room for functional innovation to evolve the functionality of lncRNAs.

Furthermore, the introduction of the “constructive neutral evolution (CNE)” mechanism may help to understand step-by-step evolution from pervasive transcription to the acquisition of functionality of lncRNAs [39]. In the CNE-like processes, new mutations are created by non-adaptive (neutral) processes. While such mutations are neutral or often slightly deleterious (“presuppressing”), they give the organisms “excess capacity” that facilitates mutational decay of the original components and leads to the generation of novel components, interactions, and functions in part through adaptive selection [43]. When adapting CNE-like processes to the interpretation of pervasive transcription, the excess capacity of pervasive transcription is considered as negative and positive cis-regulatory effects of cryptic transcription and the transcripts, including eRNAs, on nearby genes [40]. Such excess capacity may allow the original regulatory transcription sites or transcripts to mutational decay, which will later be maintained and elaborated by acquiring sequence contexts to define transcriptional directionality, termination, RNA localization, RNA structure, and functionality including both non-coding and coding functions. Under these scenarios, the production of RNA is initially sustained by selection for transcription rather than by the transcript sequence, but eventually, the latter gains function. Thus, lncRNAs can be inherently considered as byproducts of transcription, so-called “spandrel” in evolutional biology [39,44]. This evolutionary trajectory is in contrast to that of protein-coding genes that mostly originate from gene duplication [45,46]. This model is overall consistent with widespread and multifaceted local interactions of lncRNAs and eRNAs with nearby genes, which involve modulation of RNA splicing, retention of DNA- and RNA-binding proteins, and transcription [47,48,49]. In addition, in these scenarios, several sequence contexts such as U1 signal and polyadenylation signal (PAS) likely have multifaceted roles in shaping the evolution by controlling transcriptional directionality, premature termination, transcriptional elongation, nuclear localization, and connection to PAXT, beyond the conventional roles in RNA splicing and polyadenylation of mRNAs [50,51,52,53,54].

## 4. Biological Significance of Human Nuclear RNA Exosome

RNA exosome complexes are thought to be cell-essential genes required for cell viability. Reflecting that the RNA exosome targets numerous types of RNAs, experimental depletion of RNA exosome causes complex phenotypes, including alterations in translation, chromatin regulation, genome structure, R-loop formation, DNA damage response, telomere regulation, cell viability, differentiation, and cellular senescence [3,4,55]. However, it remains unclear how RNA exosome depletion causes these phenotypes through direct or indirect effects. Several missense mutations in genes encoding RNA exosome components and cofactors are linked to various rare diseases, and complete loss of the RNA exosome appears to be lethal. In this section and the following section, we summarize the physiological relevance of the RNA exosome and the pathological involvement in disease conditions with particular focus on the collection of rare diseases associated with mutations in core exosome genes and cancers.

### 4.1. Regulation of Genome Stability, Chromatin Structure, and DNA Damage Response

Depletion of the RNA exosome can drastically change the localization and accumulation of multiple RNA species. As described earlier, depletion of MTR4 and ZFC3H1 induces cytoplasmic accumulation of nuclear RNAs and perturbs translation [25,33]. In the nucleus, unwanted and excess accumulation of nuclear RNA can augment R-loop formation, DNA damage response, and genome instability. The unique roles of RNA exosome in genome stability control have been demonstrated by a series of studies in B cells, which undergo activation-induced cytidine deaminase (AID)-mediated immunoglobulin heavy-chain (IgH) class switch recombination (CSR) and somatic hypermutation (SHM) [56,57,58,59,60,61]. In B cells, cooperation between AID and the RNA exosome, together with their physical interaction, is required for normal CSR. These findings suggest that resolution of RNA:DNA hybrids (R-loop) by the RNA exosome is essential for proper mutagenesis activity of AID, chromatin configuration, and subsequent genomic rearrangement [56,57,58,59,60,61]. In addition, the RNA exosome interacts with senataxin (SETX), an R-loop-resolving RNA helicase, and depletion of SETX yields phenotypes similar to those resulting from the depletion of RNA exosome in B cells [62,63]. Consistent with this, we previously reported that depletion of EXOSC3 caused an increase in γ-H2AX and p53 levels in mouse embryonic stem cells (mESCs) [53].

A recent analysis further demonstrated that the RNA exosome was necessary for DNA repair for homologous recombination (HR) [64]. In the context of DNA damage, the RNA exosome is essential for the clearance of long non-coding RNAs (ncRNAs) transcribed at DNA double-strand breaks (DSB)-flanking sequences that form DNA-RNA hybrids. This appears to be important for the prevention of DNA hyper-resection associated with transcription, recruitment of the ssDNA-binding protein RPA to DSB sites, and assembly of HR machinery [64]. Another recent study showed a complex interplay among ncRNA, RNA exosome, and polycomb repressive complex 2 (PRC2) in the induction of heterochromatin and transcriptional silencing, suggesting expanded roles of the RNA exosome in chromatin configuration [65,66]. In addition, the RNA exosome plays important roles in suppressing other unwanted RNA molecules, including expanded hexanucleotide repeat RNA in the *C9orf72* gene in patients with frontotemporal lobar degeneration (FTLD) and amyotrophic lateral sclerosis (ALS) [67]. Conversely, arginine-rich toxic dipeptide repeat (DPR) proteins derived from repeat RNAs are suggested to impair the function of the RNA exosome [67].

### 4.2. Gene Regulation and Differentiation

Gene expression programs consist of multiple layers of feedback and feedforward regulation. This is also the case for RNA processing and RNA degradation. PABPN1 autoregulates itself by binding to an A-rich region in the 3′ UTRs of PABPN1 pre-mRNA, preventing splicing, and inducing nuclear decay [68]. Such post-transcriptional autoregulation is also observed for DGCR8, a central processor of miRNA precursors, whose mRNAs are destabilized by the Drosha-DGCR8 complex via the hairpin structures [69]. Thus, it is not surprising that widespread perturbation of RNA turnover by RNA exosome depletion perturbs spatio-temporal patterns of gene expression through direct and indirect effects, altering diverse cellular phenotypes, including differentiation, proliferation, and cellular senescence. In fact, together with dynamic changes in the expression levels of EXOSC components, the RNA exosome plays important roles in differentiation programs in various cell types, such as skin epidermal progenitors, erythrocytes, and mouse and human ESCs [21,53,70,71,72,73,74,75].

A study in mESCs has revealed that the transcripts that are differentially expressed between distinct differentiation states tend to be more exosome-sensitive in either state where the expression is lower than the other [74]. Thus, in conjunction with transcriptional regulation, the RNA exosome may contribute to both accentuation of cell type-specific transcriptional programs and rapid remodeling of transcriptomes during differentiation. Such interplay between transcription and RNA decay can be partly explained by the fact that depletion of the m6A writer METTL3 or the nuclear reader YTHDC1 protects chromosome-associated regulatory RNAs (carRNAs), including promoter-associated RNAs, enhancer RNAs, and repeat RNAs, from m6A-guided exosome degradation, and finally increases chromatin accessibility and activates transcription [76]. Consistent with this, m6A modification has important roles in rewiring pluripotency and differentiation programs in ESCs [77,78,79,80]. Furthermore, in acute myeloid leukemia (AML) cells, YTHDC1 binds to m6A RNAs and forms liquid-like condensates in the nucleus, and the condensates protect a set of PAXT target RNAs from degradation. The m6A-mediated protection of RNAs is a barrier to decreased cell proliferation, and increased cell death and differentiation of AML [81].

In addition, a recent report has shown that the RNA exosome-mediated RNA turnover has an important role in cellular senescence [82]. Senescent cells exhibit reduced turnover of multiple unstable RNAs, including 3′ extended U snRNAs and uaRNAs, short-lived mRNAs, and upregulation of the interferon-inducible genes [82]. This phenotype appears to be associated with reduced expression of RNA exosome subunits, such as DIS3L (the catalytic subunit of the cytoplasmic RNA exosome complex), EXOSC3, and EXOSC9, in several cell types. Consistently, depletion of EXOSC3 accelerated expression of multiple senescence markers and induced a senescent-like phenotype [82].

## 5. Pathological Significance of Human Nuclear RNA Exosome

### 5.1. Mutations in RNA Exosome Components and Rare Diseases

Mutations in distinct components of the RNA exosome have been linked to distinct, tissue-specific disease phenotypes, while some partly overlap [83,84,85]; such conditions are collectively called “exosomepathy”. Mutations in *EXOSC3*, *EXOSC8*, and *EXOSC9* cause several forms of autosomal-recessive neurodegenerative disease, pontocerebellar hypoplasia (PCH), namely, PCH type 1b (PCH1b), PCH1c, and PCH1d, respectively [83,84,85]. PCH1b with *EXOSC3* mutations is characterized by atrophy of the cerebellum and pons [86]. PCH1c with *EXOSC8* mutations is characterized by psychomotor deficits, cerebellar and corpus callosum hypoplasia, hypomyelination, and spinal muscular atrophy (SMA) [87]. *EXOSC9* mutations are associated with PCH1d that exhibit spinal motor neuropathy and cerebellar atrophy, while the abnormalities of the pons are modest [88]. A recent study demonstrated that mutations in *EXOSC5* are associated with a novel form of exosomepathy, characterized by developmental delays, short stature, cerebellar hypoplasia, and motor weakness [89]. In addition, the bi-allelic missense variant of *EXOSC1* was recently linked to the disease condition of PCH [90]. Although it is possible that these overlapping phenotypes are induced via distinct mechanisms in a context-dependent manner, a link between tRNA processing and other PCH types may suggest alterations in tRNAs as underlying mechanisms of abnormalities of the cerebellum and pons in these exosomepathies. Five PCH types (PCH2/4/5/6/10) are associated with mutations in genes involved in processing and modification of tRNAs, such as the tRNA splicing endonuclease (TSEN) complex (TSEN2, TSEN15, TSEN34, and TSEN54), selenocysteinyl- and arginyl-tRNA synthetase (SEPSECS and RARS2, respectively), and TSEN kinase (CLP1) [91].

In contrast to these mutations, mutations in *EXOSC2* are linked to a distinct syndrome, characterized by short stature, hearing loss, retinitis pigmentosa, and distinctive facies (SHRF), where the cerebellar atrophy is mild [92]. Given that mutations in pre-mRNA processing factors (PRPF3, PRPF4, PRPF6, PRPF8, PRPF31, SNRNP200, and RP9) have been linked to 15–20% of autosomal dominant retinitis pigmentosa, retinitis pigmentosa of *EXOSC2*-associated SHRF may be associated with misprocessing of pre-mRNAs and snRNAs [93]. As other examples, mutations in *PABPN1* have been linked to oculopharyngeal muscular dystrophy (OPMD) [94]. Mutations in the NEXT components, *RBM7* and *ZCCHC8*, have been linked to spinal motor neuropathy and familial pulmonary fibrosis with short telomere syndrome features (including bone marrow failure), respectively [95,96]. Although the underlying mechanisms appear to be different, altered expression of RBM7 is also reportedly associated with pulmonary fibrosis [97]. In addition, mutations in *SKIV2L* and *TTC37* (components of the cytoplasmic exosome cofactor, Ski complex) have been linked to syndromic diarrhea/trichohepatoenteric syndromes (SD/THES2 and SD/THES1), respectively [98,99].

The mechanistic consequences of mutations in core exosome genes remain unclear. However, mutations in the exosome subunit appear to generally impair the assembly of the RNA exosome complex and reduce overall levels of the functional complex [83,84,85]. As *EXOSC2* mutations may only mildly reduce the overall level of the RNA exosome, the degrees of disruption of the functional complex may partly explain the presence of the PCH phenotype. It is also likely that mutations in distinct core exosome genes differentially affect distinct entry paths for the RNA exosome, distinct RNA target classes, and distinct cofactors, finally leading to diverse mutation-dependent and cell type-dependent phenotypes. Furthermore, an interplay between known complex configurations and cell cycle-dependent interaction dynamics may distinguish the effects of each component on the overall behaviors of the RNA exosome [17].

### 5.2. RNA Exosome and Cancer

**Figure 3:**
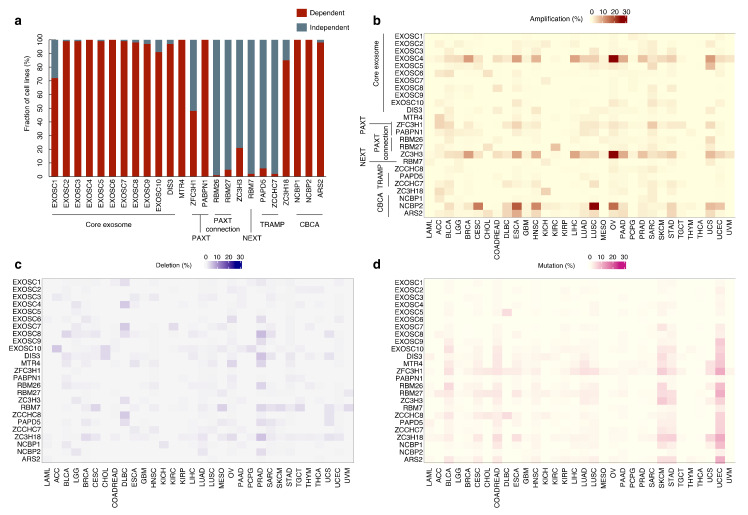
Dependencies on exosome-related genes in cancer cell lines and the alteration frequencies in human cancers. (**a**) A chart showing the percentage out of 1054 cancer cell lines, in which the dependency for a given CRISPR target gene is identified in the Cancer Dependency Map (DepMap). A cell line with a probability of dependency greater than 0.5 was considered as dependent [101]. Note that the data for a NEXT component ZCCHC8 is unavailable in DepMap. (**b**–**d**) Heatmaps showing the percentage of TCGA cases, in which (**b**) amplifications, (**c**) deletions or (**d**) mutations in given genes were evident. DepMap and cBioPortal were accessed in November of 2021. LAML, Acute Myeloid Leukemia; ACC, Adrenocortical Carcinoma; BLCA, Bladder Urothelial Carcinoma; LGG, Brain Lower Grade Glioma; BRCA, Breast Invasive Carcinoma; CESC, Cervical Squamous Cell Carcinoma and Endocervical Adenocarcinoma; CHOL, Cholangiocarcinoma; COADREAD, Colorectal Adenocarcinoma; DLBC, Diffuse Large B-Cell Lymphoma; ESCA, Esophageal Carcinoma; GBM, Glioblastoma Multiforme; HNSC, Head and Neck Squamous Cell Carcinoma; KICH, Kidney Chromophobe; KIRC, Kidney Renal Clear Cell Carcinoma; KIRP, Kidney Renal Papillary Cell Carcinoma; LIHC, Liver Hepatocellular Carcinoma; LUAD, Lung Adenocarcinoma; LUSC, Lung Squamous Cell Carcinoma; MESO, Mesothelioma; OV, Ovarian Serous Cystadenocarcinoma; PAAD, Pancreatic Adenocarcinoma; PCPG, Pheochromocytoma and Paraganglioma; PRAD, Prostate Adenocarcinoma; SARC, Sarcoma; SKCM, Skin Cutaneous Melanoma; STAD, Stomach Adenocarcinoma; TGCT, Testicular Germ Cell Tumors; THYM, Thymoma; THCA, Thyroid Carcinoma; UCS, Uterine Carcinosarcoma; UCEC, Uterine Corpus Endometrial Carcinoma; and UVM, Uveal Melanoma.

An inspection of CRISPR screening of 1054 cancer cell lines using DepMap showed that EXOSC1–10 and DIS3 (EXOSC11) are essential in most cancer cell lines (Figure 3a) [100,101]. PABPN1 and MTR4, as well as CBCA components and ZC3H18, also exhibit similar essentialness. In contrast, RBM7, a NEXT component, is essential in only a small fraction, and ZFC3H1 is essential in approximately half of cell lines. Consistent with this, an inspection of the genomic status of exosome components in TCGA database (cBioPortal) showed that loss-of-function or large deletions of the RNA exosome components are rare in human cancers (Figure 3b–d) [102,103]. Of note, at a glance, amplification of *EXOSC4* and *ZC3H3* genes are relatively frequent in certain cancer types, and clearly exhibit the co-occurrence (Figure 3b). However, these genes are located in the long arm of chromosome 8, which is well known to possess oncogenes such as *c-myc* and *c-mos* and be frequently amplified in various cancers [104,105,106]; therefore, careful evaluation is required if amplification of these genes are bona fide cancer-driver. As one exception, heterozygous *DIS3* mutations are recurrently observed in approximately 10–20% of patients with multiple myelomas and detected in AML and other cancer types [107,108,109]. Germline variants of *DIS3* have also been recently identified in familial multiple myeloma [110]. *DIS3* mutations are mainly located within the major ribonuclease domains of the protein and are thought to impair DIS3 catalytic activity. Although the mechanistic consequences of *DIS3* mutations are largely unclear, a recent report has described that cancer-associated *DIS3* mutations cause mitotic defects and genome instability without increased DNA damage and RNA processing defects in yeast [111]. In addition, another report described that DIS3-deficient B cells show accumulation of AID-mediated asymmetric nicks, alterations in SHM patterns, and increase in microhomology-mediated end-joining DNA repair. In DIS3-deficient B cells, altered mutation patterns and architectural defects of the IgH locus lead to decreased CSR but increased chromosomal translocations [61]. Although it is unclear whether these mechanisms work similarly in myeloma cells, AID and/or immunoglobulin expression may confer DIS3-dependent mechanisms specifically in myeloma pathogenesis.

A recent integrative survey of rRNA metabolism-related genes using TCGA database showed that specific upregulation of EXOSC8 expression is driven by alterations in copy number variations (CNV) and correlates with worse prognosis in colorectal cancer [112]. In addition, upregulation of EXOSC4 and EXOSC5 expression has been reported in colorectal cancer, in which upregulated expression of EXOSC5 was associated with a worse prognosis [113,114]. A correlation between high EXOSC1 expression and poor prognosis has been reported in kidney renal clear cell carcinoma [19]. Such relationships have also been observed for various core exosome components in mantle cell lymphoma [115]. Interestingly, a recent report has described that several RNA metabolic pathways, including mRNA splicing, RNA exosome-mediated RNA decay, and m6A modification, are upregulated in the Sonic Hedgehog (SHH) subgroup of medulloblastoma, which is frequently associated with mutations in U1 snRNA, suggesting the particular importance of the RNA surveillance system in this tumor type [116].

With respect to functional consequences, EXOSC9 depletion inhibits growth and survival under various stress conditions in several cancer cell lines [117]. Along with EXOSC9 depletion, depletion of EXOSC2/EXOSC4 also attenuates stress resistance and P-body formation, which are important for stress adaptation [117]. Importantly, a low EXOSC9 target signature has been shown to correlate with poor prognosis in adrenocortical carcinoma, lung adenocarcinoma, and pancreatic adenocarcinoma [117]. In addition, MTR4, whose expression is frequently upregulated in hepatocellular carcinoma, and RBM7, whose expression is upregulated in breast cancer, promote tumor progression by modulating alternative splicing-mediated metabolic switch and stabilizing *CDK1* mRNA, respectively [118,119]. A connection among m6A RNA methylation, RNA-binding proteins, and the RNA exosome has also been suggested in several reports [81,120]. Future studies may reveal the detailed contribution of the RNA exosome to cancer.

## 6. Conclusions

The present review summarizes the relationships between pervasive transcription and RNA exosome, including evolutionary crosstalk, mechanisms of RNA exosome-mediated RNA surveillance, and physiopathological effects of perturbation of RNA exosome. Co-occurrence of pervasive transcription and RNA exosome system appears to fit well with a weak selection regime, conferring evolvability to organisms based on individually weak mutations, which are promiscuous but rich sources for transcription. In addition, various phenotypes observed upon perturbation of the RNA exosome and cofactors may be viewed as functions of the RNA exosome, independent of the counterbalance system for pervasive transcription. As the RNA exosome influences genome stability, it is important to understand how the RNA exosome controls the dynamics of nuclear meso-scale structures and controls compartmentation of biochemical reactions in the nuclei. Given that various RNA molecules targeted by the RNA exosome play important roles in intracellular liquid-liquid phase separation (LLPS), the biology of biomolecular condensate and LLPS may be intimately associated with the functions of the RNA exosome [121,122]. From this perspective, the RNA exosome may not only suppress deleterious effects of cryptic transcription but also facilitate functional innovation based on multivalent weak cooperative interaction, a hallmark of LLPS-type biological reactions, through effective compartmentation of biochemical reactions [123]. A better understanding of RNA exosome involvement in pathological conditions will allow for therapeutic development targeting RNA surveillance systems.
